# Development of new *in vitro* models of lung protease activity for investigating stability of inhaled biological therapies and drug delivery systems

**DOI:** 10.1016/j.ejpb.2019.11.005

**Published:** 2020-01

**Authors:** Arcadia Woods, Teodora Andrian, Gemma Sharp, Elif Melis Bicer, Kalliopi-Kelli A. Vandera, Ayasha Patel, Ian Mudway, Lea Ann Dailey, Ben Forbes

**Affiliations:** a Institute of Pharmaceutical Science, King’s College London, 150 Stamford Street, London SE1 9NH, United Kingdom; b MRC Centre for Environment and Health and NIHR-HPRU in Health Impact of Environmental Hazards, School of Population Health & Environmental Sciences, Faculty of Life Science and Medicine, King’s College London, 150 Stamford Street, London SE1 9NH, United Kingdom; c Martin Luther University of Halle-Wittenberg, Wolfgang-Langenbeck-Str.4, 06120 Halle, Germany

**Keywords:** Respiratory, Pulmonary, Bioavailability, Albumin nanoparticles, Biologic, Peptidase, Enzyme

## Abstract

Proteases play a vital role in lung health and are critically important to the metabolic clearance of inhaled protein-based therapeutics after inhalation. Surprisingly little is known about lung fluid protease composition and there is a consequent lack of biorelevant experimental models, which limits research and development in the burgeoning field of inhaled biologics. The aim of this study was to quantify proteases in human lung fluid and to use this data to design novel *in vitro* experimental models of lung lining fluid possessing biorelevant lung protease activity for use in biopharmaceutical stability studies. As a proof of concept, these novel models were used to investigate the effect of proteolytic activity on the stability of albumin nanoparticles, a biologic nanoparticle formulation widely investigated as a pulmonary drug delivery system. Bronchoalveolar lavage fluid was collected from healthy human volunteers and proteomic analysis was used to quantify the predominant proteases. Based on these data, four new lung protease models were constructed based on: (i) trypsin as a sole protease, (ii) dipeptidyl peptidase IV, cathepsin D, cathepsin H, and angiotensin converting enzyme in ratio and concentration to mimic the protease concentration in healthy lungs. Neutrophil elastase was used to model protease activity in inflammation. Albumin nanoparticles of 100 nm diameter remained intact over 48 h in phosphate buffered saline, but were degraded more rapidly in trypsin (50% reduction in 10 min) compared to the healthy lung protease model (50% reduction in 150 min). The addition of neutrophil elastase to the healthy lung protease model resulted in a similar, but more variable degradation profile. Nanoparticle degradation was associated with concomitant appearance of small fragments and aggregates. In conclusion, we have characterised the protease concentration in the lungs of healthy humans, designed models of lung protease activity and demonstrated their utility in studying albumin nanoparticle degradation. These methods and models have wide application to study the influence of proteases in lung disease, expression of proteases in respiratory cell culture models, stability of peptide and protein-based drugs and inhaled drug delivery systems.

## Graphical abstract

**Figure:**
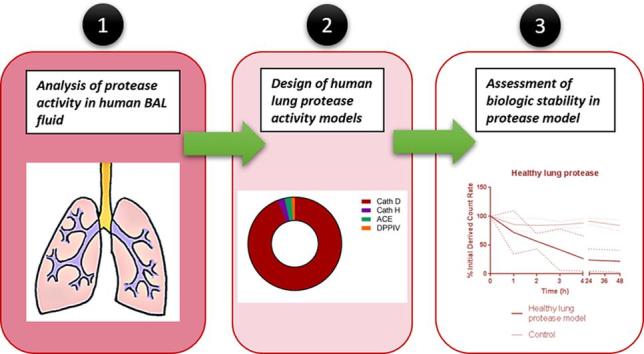

## 1 Introduction

Proteases are known to play an important role in the normal function of the healthy lungs, as well as contributing to lung pathology in various disease states [1], [2]. For inhaled biological therapies, including proteins, peptides and antibodies, lung proteases play an important role in their stability and pharmacokinetics [3]. For example, peptide therapeutics have been demonstrated to undergo significant, rapid degradation (t_1/2_ = ~6.5 min) upon incubation with lung homogenates, which contain high pulmonary peptidase concentrations [4]. The increased enzyme concentrations in the lungs associated with inflammation and diseases such as cystic fibrosis and chronic obstructive pulmonary disease (COPD) has been identified as a potential barrier for delivery of biotherapeutics by inhalation to treat respiratory diseases [5]. As an increasing number of biological therapies and drug delivery systems are in development for the inhaled route [6] there is clear benefit in establishing a model of lung protease for screening biopharmaceutical stability early in the medicines development process.

A multitude of cell-based and cell-free (liquid-only) models of the lung environment have been reported. Cell-based systems increase in complexity from simple single cell models, e.g. 16HBE14o- and Calu-3 cell lines [7], through to multi-cell 3D co-cultures (including commercial systems, e.g. MucilAir® (Alveolix) and EpiWay® (Mattek) [8], [9], [10] and advanced engineered organ-mimicking Lung-on-a-chip systems including models featuring “bioinspired respiration” motion [11]. Similarly, a variety of cell-free fluids designed to mimic the respiratory lining fluid have been developed for pharmaceutical development purposes (e.g. dissolution profiles, stability and toxicity studies) which increase in complexity from physiological-salt solutions e.g. Gamble’s Solution [12] through to multi-phase mixtures including salts, proteins and lipids [13]. Various studies have been reported which characterise lung protease activity in respiratory cell cultures [14] and to investigate the effect of lung proteases on inhaled drug integrity and transport [15]. Baginski et al. [16] have come closest to simulating degradation conditions within the lung, using a simple mixture of proteases to mimic lung conditions. However, to the best of the authors’ knowledge, no standardised, data-informed and readily prepared model of lung protease activity has so far been reported.

Numerous proteolytic enzymes are expressed in the lungs, although the evidence for the absolute concentrations and/or enzymatic activities of these is contradictory [17]. The aim of this study was to measure protease concentration in human lung lavage samples and use this data to design models of human lung protease activity, and to investigate the stability of a model biological system, albumin nanoparticles, when exposed to the models.

Albumin nanoparticles were selected as the test system for a proof-of-concept investigation of particle stability in the protease models. Albumin nanoparticles have received increasing interest as a controlled release formulation for inhaled use. They have been demonstrated to be well tolerated in the mouse lung, and demonstrate a long residence time after deposition (>48 h) [18]. This has been shown to translate to improved treatment outcomes when used as a carrier, for example to deliver tacrolimus for treatment of pulmonary fibrosis [19], and a doxorubicin-TRAIL co-formulation for treatment of lung cancer [20]. An important stage in the development of this formulation for use in the clinic will be to understand the fate of the nanoparticle carriers following drug release. We hypothesise that as albumin is a substrate of many proteolytic enzymes, this biopharmaceutical formulation will be liable to degradation in the newly developed protease model.

A quantitative study of proteases in healthy human lungs was performed by the proteomic analysis of bronchoalveolar lavage fluid (BALF) samples collected from healthy human volunteers. Proteomic data was used to prepare four different models of lung protease for further investigation: i) trypsin, ii) healthy lung protease, iii) elastase and iv) inflamed lung protease. Neutrophil elastase was employed to model the conditions of protease during lung inflammation. This potent, broad-activity proteinase is well-established as a key mediator in the lung inflammatory pathway and due to its activity against a broad range of substrates is associated with substantial damage to the cells and tissues of the lungs when levels become elevated [21]. In cystic fibrosis (CF), increased neutrophil infiltration to the lungs along with dysfunction in protease-regulatory mechanisms can lead to high local elastase concentration [22], which in turn can result in degradation of extracellular matrix and airway remodelling. Degradation of albumin nanoparticles following protease exposure was monitored using dynamic light scattering through analysis of derived count rate (kcps) and the particle size distribution. The effects of protease exposure were compared to the effect of incubation in phosphate-buffered saline (PBS) as a control.

## 2 Methods

### 2.1 Materials

Bovine serum albumin (essentially IgG-free, low endotoxin) and dipeptidyl carboxypeptidase I/Angiotensin Converting Enzyme (ACE) (from rabbit lung lyophilized powder; 2–6 units/mg protein; modified Warburg-Christian) were purchased from Sigma-Aldrich (Dorset, UK). Cathepsin D (recombinant human) was purchased from Cambridge BioScience (Cambridge, UK). Dipeptidyl-peptidase IV (DPP4) was purchased from Source BioScience (Nottingham, UK). Trypsin-Ethylene diamine tetraacetic acid (EDTA) 0.25% w/v in Hanks Balanced Salt Solution (HBSS) was purchased from VWR (Bedfordshire, UK). Cathepsin H (active, human) and neutrophil elastase (recombinant human; produced in yeast) were purchased from Generon (Slough, UK). Phosphate buffered saline (PBS) tablets were purchased from Oxoid (Basingstoke, UK). PBS (1x) was prepared as per the manufacturer’s instructions using ultrapure water. Disposable, solvent resistant micro cuvettes were purchased from Malvern Panalytical (Malvern, UK).

### 2.2 Proteomic analysis of human bronchoalveolar lavage samples and design of the lung protease models

The proteomic analysis of human airway lavage fluids derived from healthy volunteers was approved by the local ethics committee at the University Hospital, Umea, Sweden, in accordance with the Declaration of Helsinki. Informed written consent was obtained from all subjects prior to inclusion into the study. Bronchoalveolar lavage (BAL) fluid was collected from five healthy subjects (n = 27 ± 2 years, 4 females/1 male) using established methods [23]; additional detail is provided in the supplementary material. Following removal of mucus and cells, the cell free lavage was concentrated using 9 K MWCO iCON Pierce concentrators (Thermo Scientific, UK), with the subsequent filter retentates resuspended at equal concentrations and separated using 1D page, prior to band excision, trypsin digestion and protein extraction. The final peptide mixtures were analysed with an automated nanoLC MS/MS system, followed by protein identification using Mascot (Matrix Science, London, UK; version 1.3.0.339) and X! Tandem (The GPM, thegpm.org; version CYCLONE (2010.12.01.1)), with protein abundance calculated as the sum of the three most intense peptide precursor ions. Full details of this proteomic pipeline are presented in the supplementary material. Of the 119 identified proteins, 13 were classified as proteases based on their gene ontologies (see Table s1, supplementary material). Of these species, 4 were confirmed as proteolytic enzymes with previously reported presence in the lung lining fluid (epithelial lining fluid, ELF, or BAL) (Table 1). The arbitrary relative abundance units were converted to mass values by normalisation to a well-characterised protein concentration also identified during the analysis. Lactoferrin was identified in BAL samples with a relative abundance of 1.30 × 10^9^. A literature value of lactoferrin concentration (0.1 mg/mL) was used to convert the relative abundance values of the proteolytic enzymes to mass based concentrations [24] which were used for the protease model construction (Eq. (1)).

(1) $$\mathrm{Concentration}\;of\;proteolytic\;species,\;x\left(\frac{\mu g}{\mathrm{mL}}\right)=\frac{\mathrm{Relative}\;abundance\;\left(x\right)}{\mathrm{Relative}\;abundance\;\left(Lactoferrin\right)}\times 100$$

**Table 1:** Proteases identified in lung lining fluid and conversion to mass-based concentration values.

Non-Protease	Relative Abundance	Concentration (µg/mL)
Lactoferrin	1.38E + 09	100

Proteolytic Component	Relative Abundance	Relative concentration (µg/mL)

Dipeptidyl peptidase 4	1.64E + 07	1.19
Dipeptidyl carboxypeptidase I/ Angiotensin-converting enzyme	2.86E + 07	2.07
Pro-cathepsin H	2.79E + 07	2.02
Cathepsin D	1.10E + 09	79.9
Lower abundance proteolytic components	7.91E + 07	5.73
	SUM	90.94

### 2.3 Preparation and characterisation of albumin nanoparticles

Albumin nanoparticles were prepared using the desolvation method as reported previously [25]. In brief, 50 mg of bovine serum albumin (BSA, essentially IgG-free, low endotoxin, Sigma, UK) was dissolved in 0.5 mL of 0.01 M Tris HCl (pH 8.9) and the pH adjusted to 9 with the addition of NaOH (12.5 μL, 1 M). Ethanol (2.0 mL) was added dropwise to the stirred protein solution at a rate of 1 mL/min. Nanoparticles were cross-linked by addition of 8% vol/vol glutaraldehyde in water (4 μL) followed by stirring overnight. Excess glutaraldehyde, NaOH, ethanol and unreacted BSA were removed by four cycles of spin filtration (100 kDa MWCO Amicon® ULTRA centrifugal spin filters, Merck-Millipore, USA) and buffer exchange with freshly prepared PBS. Concentration of BSA nanoparticles in the purified suspension was assessed gravimetrically.

Particle size and polydispersity (Polydispersity Index, PdI) were assessed using dynamic light scattering (DLS) with the Zetasizer Nano Series ZS instrument (Malvern Panalytical, Malvern, UK). Particles were diluted 1:10 with PBS prior to measurement. Size measurements were performed in triplicate using a scattering angle of 173° and temperature of 25 °C.

### 2.4 A dynamic light scattering method for quantification of enzyme-mediated particle degradation

Degradation of the model biopharmaceutical system, albumin nanoparticles, was assessed using DLS using two outputs: i) derived count rate (kilo counts per second, kcps) and ii) particle size distribution. The derived count rate is defined as the number of photons reaching the detector and represents the light scattering intensity detected by the instrument. Count rate has been demonstrated to be proportional to nanoparticle concentration [26], and thus can be used as a tool to monitor the proportion of intact nanoparticles remaining in suspension following incubation in protease models. To validate the relationship between albumin particle concentration and count rate, five calibration suspensions were prepared at concentrations of 0.125, 0.25, 0.5, 1.0 and 2.0 mg/mL with PBS as diluent. Calibration suspensions were diluted 1:10 with PBS prior to measurement and analysed in triplicate at 25 °C using the Zetasizer Nano Series ZS. Five replicates of each standard curve were measured.

### 2.5 Particle degradation studies in four models of lung protease activity

#### 2.5.1 Trypsin model

**Fig. 1:**
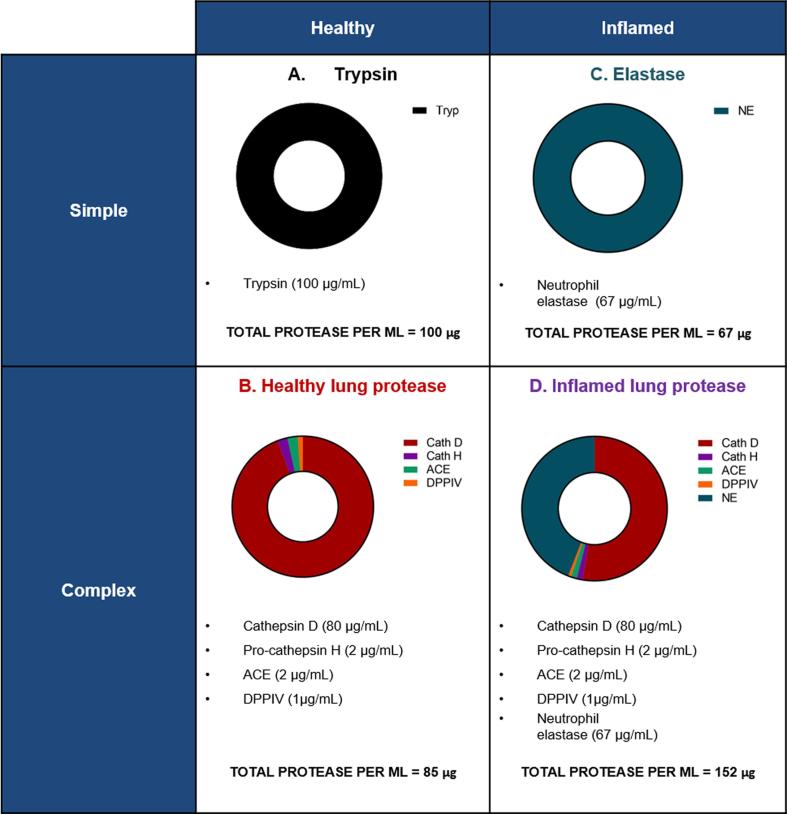
Composition of the four *in vitro* models designed to represent the protease activity in the lungs. Healthy human lung models were based on (A) trypsin (B) a cocktail of the four most abundant proteases recovered from human alveolar bronchial lavage fluid. Inflamed models were based on (C) neutrophil elastase, (D) the cocktail of healthy lung proteases from (B) plus elastase from model C. The total amount of protease in models A, B, C and D was 100 µg/mL, 86 µg/mL, 67 µg/mL and 152 µg/mL, respectively.

Based on the BAL fluid proteomics analysis, a total protease concentration of 0.091 mg/mL was calculated (see Section 3.1). The first protease model prepared was a trypsin model representing the total protease concentration in the BALF (100 µg/mL). The particle concentration (1.6 mg/mL) was selected to approximate the particle concentration in the epithelial lining fluid at the no observed adverse effect level (NOAEL) reported in a lung biocompatibility study for this formulation [18]. Albumin nanoparticles (1600 µg) were incubated with trypsin (100 µg) and PBS (Fig. 1A) at 37 °C and samples removed at pre-determined intervals over 48 h. Particles incubated in PBS alone at 37 °C were used as controls. Aliquots (10 μL) of the enzyme-particle mixture were removed at 0, 0.5, 1, 2, 4, 24 and 48 h, diluted in 90 μL of PBS and analysed immediately using the Zetasizer Nano Series ZS (Malvern).

#### 2.5.2 Healthy lung protease model

The second model was prepared from the four most-abundant proteases identified in human BALF as a model of protease in the human lung. For inclusion in the model, proteolytic species identified in BALF proteomic analysis had to be i) a confirmed protease and ii) have been previously reported to be present in respiratory tract lining fluid and/or mucus. The four proteases chosen for the model (cathepsin D, cathepsin H, dipeptidyl carboxypeptidase I/ angiotensin-converting enzyme (ACE) and dipeptidyl peptidase IV) all satisfied these criteria.

Prior to the degradation study, cathepsin D was activated according to manufacturer’s instructions. In brief, cathepsin D (125 µL) was incubated with 12.5 µL of buffer (1 M NaOAc, 2 M NaCl, pH 3.8) at 37 °C, for 30 min. The resulting activated enzyme solution was used to prepare the healthy protease model. Albumin nanoparticles were incubated with the pre-warmed enzymes in PBS at 37 °C at a ratio of 1600 µg particles/ 85 µg protease (Fig. 1B). Aliquots (10 μL) were removed from the degradation mixtures at 0, 1, 2, 3, 4, 24 and 48 h. Samples were diluted 1:10 with PBS and immediately analysed using the Zetasizer Nano Series ZS (Malvern) in triplicate. Particles incubated in PBS alone (48 h, 37 °C) were also analysed at the same time points as a control.

#### 2.5.3 Elastase and inflamed lung protease models

Neutrophil elastase was chosen to model the protease conditions in the lung during inflammation. In this study, a previously reported literature concentration of 2.3 μM (67 μg/mL) neutrophil elastase measured in BALF samples taken from CF patients was chosen [27] for the model. This was chosen as a conservative estimate of the concentration present during lung inflammation, as the BALF samples were taken from patients with mild lung disease at the time of collection. Initially, degradation of the test albumin nanoparticle system following exposure to NE-alone was investigated. Albumin nanoparticles were incubated with neutrophil elastase in PBS at a ratio of 1600 μg particles/67 μg enzyme (Fig. 1C) and degradation assessed over 24 h at 37 °C.

An inflamed lung protease model was prepared using the healthy model with addition of neutrophil elastase. The healthy lung protease model was prepared as described above, including pre-activation of cathepsin D. Neutrophil elastase (100 μg/mL stock) was added to the protease mixture to a final ratio of 1600 μg particles/67 μg enzyme (Fig. 1D). Albumin nanoparticles were again incubated with the pre-warmed enzymes in PBS at 37 °C, and samples removed and analysed as per the trypsin and healthy protease model.

## 3 Results

### 3.1 BALF analysis and identification of major proteases

BALF analysis data identified 14 proteolytic species, of which the four most abundant confirmed proteases (cathepsin D, cathepsin H, dipeptidyl carboxypeptidase I/ angiotensin-converting enzyme (ACE), dipeptidyl peptidase IV (DPP4)) were chosen to prepare the lung protease model. Relative abundance values as calculated from proteomic data were converted to mass-based concentrations using Eq. (1) as described above, resulting in concentrations of 79.9 μg/mL cathepsin D, 2.02 μg/mL pro-cathepsin H, 2.07 μg/mL ACE and 1.19 μg/mL DPP4 (Table 1). Nine further proteolytic species were identified with a combined concentration of 5.73 μg/mL. The total concentration of proteolytic components identified in the BALF was 90.94 μg/mL. This data was used to inform the trypsin, healthy lung protease and lung inflammation protease models.

### 3.2 Characterisation of albumin nanoparticles as the test biopharmaceutical system

**Fig. 2:**
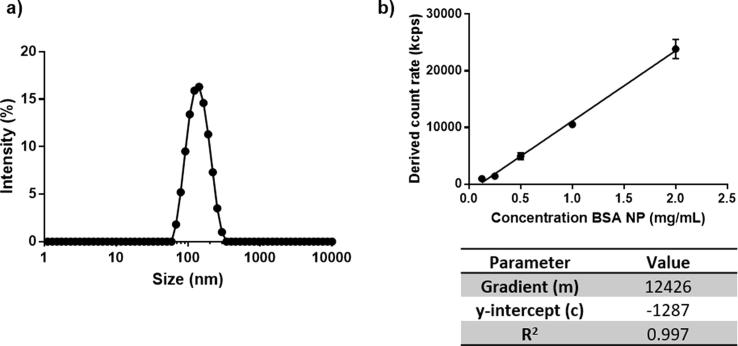
Characterisation of albumin nanoparticles used as test biopharmaceutical for degradation studies. Fig. 1a depicts representative particle size distribution obtained for albumin nanoparticles at 10x dilution in phosphate-buffered saline at 25 °C. Fig. 1b represents linear relationship between derived count rate (kcps) vs albumin nanoparticle concentration. Data represent mean ± SD (n = 5).

Albumin nanoparticles to be used as the test biopharmaceutical system for protease stability studies were manufactured in house and characterised before use. Albumin nanoparticles were monodispersed with a mean particle diameter of 127.6 ± 2.7 nm and narrow size distribution (P.d.I 0.107 ± 0.021) (Fig. 2a). Particles were found to be stable upon storage at 4 °C for up to 83 days (data not shown).

Protease-mediated particle degradation was monitored using the derived count rate (kcps) obtained during photon correlation spectroscopy measurements. Count rate analysis is a simple rapid technique allowing *in situ* assessment of particle degradation which has been previously demonstrated to correlate with more traditional dissolution and drug release studies to monitor particle breakdown [28].

To validate the relationship between count rate and albumin nanoparticle concentration, a standard curve was prepared which demonstrated a linear relationship (R^2^ = 0.997) between albumin nanoparticle concentration and count rate between concentrations of 0.125–2 mg/mL (Fig. 2b). This demonstrated that the analytical technique was capable of monitoring changes in albumin particle concentration caused by enzyme-mediated degradation in the experimental range.

### 3.3 Count rate as an indicator of protease-mediated degradation

**Fig. 3:**
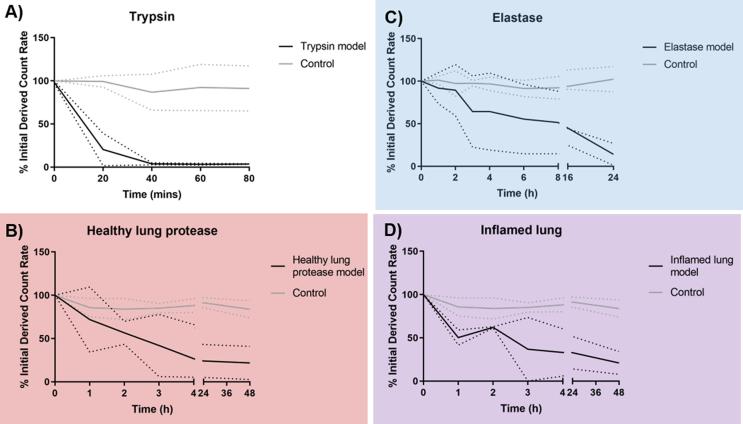
Degradation of albumin nanoparticles following exposure to the protease models compared to phosphate buffered saline control at 37 °C for different durations up to 48 h. The protease models were A trypsin, B) healthy lung protease model, C) elastase, and D) inflamed lung protease model. Degradation was illustrated by the reduction in derived count rate measured using dynamic light scattering. Dotted lines (···) represent the standard deviation from the mean. Data represent mean ± SD (N = 3–6).

Albumin nanoparticles were employed as a test biopharmaceutical to investigate stability to proteolytic degradation in the four protease models: trypsin, healthy lung protease, elastase and inflamed lung protease. Albumin nanoparticle count rate was demonstrated to undergo significant, rapid reduction following incubation in the trypsin model (Fig. 3) with 80 ± 19% count rate drop within the first 20 min of exposure reducing further to ~96% by 40 min. This was indicative of a rapid, significant reduction in intact particle number following exposure to the trypsin protease model.

Incubation of albumin nanoparticles in the healthy lung protease model also resulted in a significant reduction in count rate, albeit at a slower rate and with greater variability than the trypsin system. By 4 h, count rate had reduced by 73.3 ± 39.4% compared with no significant decrease following incubation in PBS alone. The count rate remained at a similar level for the remaining 44 h of the study, indicating no further significant change. Of interest were the large sediments detected by visual observation at timepoints >3 h, which were also reflected in particle size distributions (see section 3.4). A statistically significant decrease in the count rate was confirmed following exposure to the healthy protease model over 48 h compared to incubation in PBS alone (P < 0.001, 2-way ANOVA).

Neutrophil elastase degradation of albumin nanoparticles was investigated alone (elastase model) and in combination with the healthy protease model to mimic the effect of lung inflammation (inflamed lung protease). This enzyme was selected as neutrophil elastase is widely reported to be expressed at higher levels in the lung than normal during inflammation [2]. Degradation in the elastase model demonstrated high levels of variability in contrast to trypsin, however was still observed to take place. When incubated with NE alone, albumin nanoparticles underwent a gradual breakdown with 48.6 ± 36.8% reduction in count rate over the first 8 h exposure, with a further significant reduction down to 14.0 ± 13.0% of the original count rate by 24 h. Protease-mediated count rate reduction was again significantly increased compared to 24 h incubation in PBS alone (P < 0.05, 2-way ANOVA).

Interestingly, the addition of neutrophil elastase to the healthy protease model (inflamed lung protease model) did not significantly increase the rate of degradation despite the increased protease concentration in this system.

### 3.4 Particle size changes during protease-model mediated degradation

**Fig. 4:**
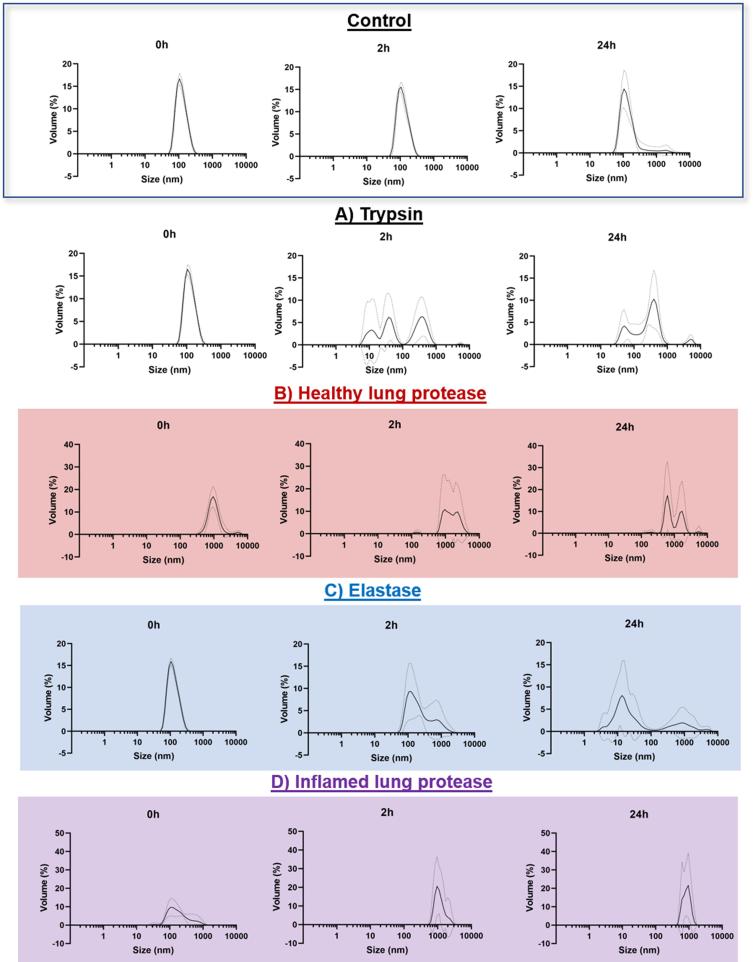
Degradation, aggregation and increase in polydispersity of the test system, albumin nanoparticles, following exposure to the protease models (A: trypsin, B: healthy lung protease, C: elastase and D: inflamed lung protease) for 24 h at 37 °C. Data represent mean ± SD (N = 3–6) and illustrate particle size distributions determined by dynamic light scattering and include a control of albumin nanoparticles in phosphate buffered saline.

Changes in particle size distribution supported the findings derived from count rate analysis and showed a clear difference in the stability of the albumin nanoparticle test system following incubation in the four protease models compared to PBS alone. Other than the appearance of a small number of aggregates at 24 h onwards, no significant increase in size or polydispersity was observed throughout the 48 h incubation in PBS at 37 °C (Fig. 4). In contrast, significant and distinct changes were observed following incubation in the four different protease models.

Incubation in the trypsin model resulted in the smallest change to the particle size distribution. Three peaks were present at 2 h onwards (10 nm, 40 nm and 400 nm) suggesting the presence of both smaller particle fragments and larger aggregates because of protease exposure. However, it should be noted that due to the significant drop in count rate observed (Fig. 3), these particles represent a very small number relative to the starting population.

Distinct differences in stability of the albumin system were observed for the remaining three protease models. Incubation in the healthy protease model resulted in immediate appearance of large aggregates (1000 nm) at 0 h. By 2 h, the particle size distribution showed a highly variable, multimodal particle population, resulting in a large standard deviation (33%) in mean particle size (Z av at 2 h: 2462 ± 1319 nm). Incubation of albumin nanoparticles in the elastase model resulted in the broadening of the peak and presence of aggregates in the distribution (800 nm) by 2 h. The average particle size and standard deviation increased to 163 ± 65 nm, a relatively modest change compared to the significant aggregation detected for the healthy protease cocktail. By 24 h, a significant peak was also observed at 15 nm, suggesting the presence of small fragments as well as particle aggregates.

Incubation in the inflamed lung protease model initially did not induce the same extent of significant aggregation observed for the healthy model, with a peak still visible at 120 nm. However, by 2 h only the larger aggregates were evident in the particle size distribution (average diameter 2680 ± 509 nm). Polydispersity (P.d.I.) increased from 0.156 ± 0.081 at 0 h to 0.565 ± 0.191 at 2 h, again showing a tendency to increase in polydispersity following incubation in the protease models.

## 4 Discussion

The ability to predict behaviour in the lung environment is a crucial stage in the development of any inhaled product. For biopharmaceuticals, the ability to predict stability to protease in the lungs is of particular importance. Whilst *in vitro* models of the pulmonary environment have increased significantly in their complexity and ability to predict performance *in vivo* [13], there is no generally accepted synthetic model to represent protease activity in the lungs. This study established four data-informed models of human lung protease activity, and employed these models in a proof-of-concept study to investigate the stability of albumin nanoparticles, an emerging biomolecular drug carrier formulation that offers the possibility for controlled release in the lungs [18], [20]. The changes to particle size, polydispersity and stability observed would not have been predicted following incubation in simple buffer systems.

Proteomic analysis of BALF from healthy human volunteers was used as the basis for the four protease models used in this study. The analysis identified a total protease concentration of 90.9 µg/mL. The four most abundant proteases were cathepsin D, cathepsin H, ACE and DPPIV (Table 1), all four of which have been associated with lung tissues and/or fluid. Cathepsin D, the most abundant enzyme, is an aspartic protease which has been identified in human lung epithelial cells and in high abundance within alveolar macrophages [29]. Cathepsin H, a cysteine protease, has been shown to be expressed in type II alveolar cells and alveolar macrophages [30], whilst ACE and DPPIV have both been quantified in human epithelial lining fluid [31], [32]. Very little information has been published with respect to concentrations of proteases within lung lining fluid. Juillerat-Jeanneret and colleagues [31] reported lower enzymatic activity of DPPIV in the lung lining fluid from healthy volunteers in terms of enzymatic activity per mL compared to that of ACE, however the mass ratio was not reported. Rossman and colleagues [33] reported mass-based cathepsin D concentration of 380 μg protease per 10^6^ in alveolar macrophages.

The simplest protease model was based on trypsin at the total protease concentration in the lungs calculated from the proteomic data (100 µg/mL). Incubation in the trypsin model resulted in rapid degradation of albumin particles (50% reduction in count rate within 10 min; Fig. 2a) as indicated by drop in count rate measured from dynamic light scattering. This concords with a report that albumin nanoparticles (1 mg/mL particles to 50 μg/mL trypsin) were degraded completely within 90 min, with the rate of degradation inversely proportional to the glutaraldehyde crosslinking [34]. The particles used in our study were ~15% crosslinked compared to 40% in the reported study, which may explain the faster rate of degradation observed. The degradation rate of albumin nanoparticles following incubation in the healthy lung protease model was lower compared to trypsin, i.e. 50% reduction within 150 min and 73% reduction in count rate by 4 h (Fig. 2b). Thus, we have managed to determine the stability of albumin nanoparticles in a biorelevant multi-protease ‘cocktail’ that combines enzymes in amounts and proportions guided by reference to their ratio and concentration in BAL.

Cathepsin D has been reported to degrade albumin within macrophages [35], and as the major component of the lung protease model it is highly likely that it contributes significantly to nanoparticle breakdown. The optimal pH for degradation of albumin by cathepsin D has been reported to be 3.5–4.5, but interestingly in the same study, this optimal pH was shifted towards 5–5.4 if the protein was pre-treated with formaldehyde [36]. As albumin nanoparticles are stabilised by cross-linking with a related polymerizing reagent, glutaraldehyde, the formulation of the protein into particles may change the optimal conditions for degradation. Less is known about the degradation of albumin by the other components of the model. DPPIV may be able to partially degrade albumin, although the precise mechanism is unclear [37]. Cysteine proteinases are known to contribute to intracellular albumin degradation [38], therefore it is reasonable to hypothesise that cathepsin H may also play a role in albumin nanoparticle breakdown in the model. By contrast, albumin may inhibit the activity of ACE at its physiological concentration (32–64 mg/mL) [39].

Two models were designed to mimic an inflammatory environment in the lungs. Neutrophil elastase is a potent, broad-substrate enzyme which is released during environmental or pathogen-stimulation of neutrophils in the airway [40]. Albumin is a substrate of neutrophil elastase, with degradation beginning within 30 min exposure [41]. Our study demonstrates that elastase alone can breakdown albumin in nanoparticle form. An inflamed lung protease model was prepared by supplementing the healthy protease cocktail with neutrophil elastase. Interestingly, the addition of NE did not result in an increase in the degradation rate of albumin nanoparticles (Fig. 2). The reasons for this are not clear, however it is reasonable to hypothesise that the failure to increase activity may result from complex interactions between the different proteolytic components.

To the best of the authors’ knowledge, this study represents the first attempt to prepare a data-informed model of human lung protease conditions and as such the model employed is a relatively simple system. However, the model could be developed further to include the addition of antiproteases known to be present in the lung, for example alpha-1 anti-trypsin [42], as it would be of interest to see whether these biomolecules affect the kinetics of protease-mediated degradation. Future work might also explore the role that individual proteases play in degradation, whether the proteases may interact (e.g. protease/protease degradation) and the effect of pH. Whilst it was not in the scope of this study, it would be of significant interest to characterise the proteases present within the lung lining fluid of patients with specific disease states (e.g. cancer, asthma, respiratory infection), and to use this data to prepare models applicable to the testing of a specific therapeutic with its most appropriate protease environment (e.g. inhaled antibody for asthma treatment [43] tested in asthmatic lung protease model).

Count rate analysis using dynamic light scattering was used for rapid, *in situ* monitoring of particle stability following incubation in the protease models. Methods which employ light scattering to monitor changes to particle stability have been reported previously. Anhalt and colleagues [28] reported the use of *in situ* monitoring of nanocrystal dissolution using count rate analysis from the Zetasizer (Malvern Panalytical). They demonstrated that the dissolution kinetics measured using this technique correlated with conventional dissolution methods measuring drug release. For our protease models, it was extremely beneficial to remove the need to separate the enzyme and nanoparticles before measurement at very early time points (e.g. in the trypsin model) and thus *in situ* measurement of the count rate analysis was highly advantageous. Another light scattering technique (turbidimetric analysis) has been employed previously to monitor the degradation of albumin nanoparticles upon protease exposure [34] and reported comparable kinetics to our study. For other inhaled biologics, e.g. enzymes or monoclonal antibodies, it would be of interest to include functional assays to assess the biological activity as well as physicochemical stability over time.

Physical stability may affect the uptake, bioavailability, pro-inflammatory potential and even activity of a biopharmaceutical. In the case of the albumin nanoparticle test system, both small fragments (<100 nm) and large aggregates (2−5 μm) were observed following protease exposure. Whilst the presence of small fragments is intuitive because of degradation, the precise mechanism of aggregation following protease exposure is not known. Lin et al. [44] have previously reported that the degradation of albumin nanoparticles by trypsin is likely to occur via surface erosion. As the surface charge of albumin nanoparticles has been shown to be highly important for their physical stability [45], we hypothesise that the changes occurring at the surface during enzymatic metabolism may result in destabilisation and could be the cause of the aggregation observed in this study.

The change in particle size distributions following protease exposure provides interesting insight into the possible fate of these particles in the lung *in vivo*. We have previously reported that following administration of In-111 labelled albumin nanoparticles to the lungs of mice, a significant fraction of albumin-associated In-111 activity was quantified within the lung tissue from 4 h post-deposition, along with increased activity in BAL cells compared to deposition of albumin in its solution form [18]. Micron-sized particles (~2–3 μm) have been demonstrated to undergo increased alveolar macrophage uptake compared to smaller particles [46], which may suggest a possible mechanism for the accumulation of albumin-associated activity in BAL cells after lung deposition. Conversely, degradation of albumin nanoparticles into smaller peptide and amino acid fragments may facilitate their uptake into lung tissue, as absorption from the lung has been demonstrated to be inversely proportional to molecular weight [47].

This study employed albumin nanoparticles as the first test system to be applied to the four lung protease models. As a carrier system, the degradation of albumin nanoparticles is primarily of interest in order to predict drug release rates, lung retention and toxicity potential. However, these models would also be of significant interest in future for pharmacologically-active biomolecules, including antibody-based therapies and therapeutic enzymes. For these biomolecules, protease-mediated degradation could affect the function of the therapeutic and thus render some inactive. Recent studies have suggested that engineering approaches, e.g. addition of polyethylene glycol (PEG) to biomolecules (e.g. recombinant human deoxyribonuclease I (rhDNase)) can improve their stability to proteolysis, and thus improve their activity [48]. Changes to protein structure, including aggregation and degradation, are known to modulate the immunogenicity of the biomolecules [49] and so understanding protease stability is an important element of predicting the safety of biotherapeutics. In this context, the development of a standardised, well-characterised model of lung protease activity is of significant value in enabling the screening of new biomolecules for their stability, and design and testing of protective strategies to improve resistance to protease activity before beginning *in vivo* pharmacokinetic and pharmacodynamic studies. We present in this study an important first step in the development of such a model for widespread application in the inhaled biopharmaceutical field.

## 5 Conclusions

This study reports four data-informed *in vitro* experimental models of human lung protease activity. We have demonstrated the significant role that proteases can play in the stability of biotherapeutics designed for delivery to the lungs (albumin nanoparticles) and have illustrated the use dynamic light scattering to provide *in situ* monitoring of protease-mediated degradation using both the count rate and particle size distribution outputs. As *in vitro* models of respiratory tissues improve in their sophistication and ability to predict *in vivo* performance, this study has highlighted a pressing need to ensure that the effects of protease are considered in predictive models. The data presented here demonstrates the feasibility of creating simulants that model lung protease activity realistically and how these simulants can be employed to investigate stability in the lung environment during inhaled biotherapeutic development. These are key first steps in the development of fit-for-purpose, validated and predictive models of lung protease which will be of significant value in the pre-clinical development of biopharmaceuticals for inhalation.
