# Supplementary material for: Development of new in vitro models of lung protease activity for investigating stability of inhaled biological therapies and drug delivery systems
Source: Eur J Pharm Biopharm. 2020 Jan;146:64–72. doi: 10.1016/j.ejpb.2019.11.005 (PMC6963770; doi:10.1016/j.ejpb.2019.11.005)
Supplement: Supplementary Data 1 [file mmc1.docx]

# 6. Supplementary material

## 6.1 Supplementary methods

**Bronchoscopy procedure**

Bronchoscopy was performed, following an overnight fast. A flexible video bronchoscope (Olympus BF IT240, Tokyo, Japan) was inserted through the mouth with the subject in the supine position. Pre-medication with atropine (1 mg) was given subcutaneously 30 minutes prior to bronchoscopy to reduce airway mucous secretion, with Lidocaine (5% and 1%) sprayed onto the airways to achieve topical anaesthesia. A low volume bronchial wash was performed by infusing two aliquots of 20 mL sterile sodium chloride (NaCl), pH 7.3 at 37^o^C into the lingular or middle lobe, which was gently aspirated after each infusion. These recovered aspirates were kept as separate aliquots on wet ice. Bronchoalveolar lavage (BAL) was performed immediately after these small volume infusions by the instillation and immediate aspiration of 3 consecutive aliquots of 60 mL saline. The recovered aspirates were pooled and placed on wet ice prior to transport to the laboratory for processing. All lavage samples were passed through a nylon filter (pore diameter 100µm) and centrifuged at 400g for 15 minutes at 4˚C. The cell-free lavage derived from the BAL procedure was then aliquoted into 50mL aliquots and stored at -80° prior to down steam processing for proteomic analysis.

**Proteomic analysis**

Large volume cell free BAL samples from five healthy young adults (27±2 years, 4 females/1 male) were selected for concentration using 9K MWCO iCON Pierce concentrators (Thermo Scientific). Twenty ml of each BAL sample was loaded into iCON concentrators prior to centrifugation at 4,000 rpm at 4^o^C, for 15-minute cycles until complete. The resultant filter retentate, corresponding to lavage components more than 9kDa was then resuspended in HBSS. The highly abundant plasma protein albumin was removed from the concentrated BAL samples, using SwellGel Blue Albumin Removal Discs (Pierce) according to the manufacturer’s instructions, prior to sample separation using 1D-page.

Volumes equivalent to 100 µg protein per sample were lyophilized in a SpeedVac (Eppendorf), resuspended in sample loading buffer (63 mM Tris HCl, 10% glycerol, 2% SDS, 0.0025% bromophenol blue, pH 6.8) in 50mM DTT (Sigma) and denatured at 87^o^C for 5 minutes prior to SDS-PAGE. Samples were loaded into 4-12% Precast Bis Tris gels (NuPAGE, life technologies) using MOPS SDS running buffer (NuPAGE, life technologies) in an XCell sure lock gel electrophoresis chamber (Life Technologies). HiMark™ Pre-Stained HMW Protein Standards were used as a molecular weight markers. A potential of 50v was applied for 15 minutes, increased to 150v and run for 1 hour. Gels were removed and bands stained using InstantBlue Coomassie stain (Expedeon) on a rocker for one hour, finally gels were left to rinse with water overnight. Ten protein bands were excised per sample and washed sequentially with dH_2_O. Excised bands were cut into cubes (ca 1mm^3^), transferred into LoBind eppendorff tubes and centrifuged for 5 minutes on a bench top microcentrifuge. Fifty µl of Acetonitrile was then added to shrink the gel pieces and the samples dried in a Speed-Vac. To reduce the protein, gel pieces were subsequently swollen with 50 µl ul DTT, 0.1M ammonium bicarbonate for 30 minutes at 56 ^o^C. Following this step, 50 µl iodoacetamide, 0.1M ammonium bicarbonate was added and samples incubated at room temperature for 20 minutes in the dark. All steps were performed on an orbital shaking platform. To destain gel pieces, residual liquid was removed, and gel pieces washed with 200ul 0.1M ammonium bicarbonate for 15 minutes on an orbital shaker (Benchmark). Residual liquid was then removed, and acetonitrile added to shrink gel pieces for ten minutes. Samples were then dried for 30 minutes by Speed-Vac.

Dried gel pieces were then saturated with Trypsin digestion buffer (13 ng/ml Trypsin (Promega), 50mM ammonium bicarbonate) for 45 minutes on ice. Finally, 10µl of this digestion buffer was added to samples and they were incubated at 37^o^C overnight. To ensure optimal protein extraction, digested samples were centrifuged and incubated with acetonitrile at 30 ^o^C for 30 minutes. The supernatant then transferred to a new LoBind ependorff tube, whilst 50 µl of 1% formic acid was added to the digested gel pieces and incubated for a further 20 minutes. This supernatant was then transferred to the LoBind ependorff tube described above and the process repeated. One-hundred and fifty µl of Acetonitrile was then added to shrink gel pieces and the supernatant again transferred. The supernatants were lyophilized in a SpeedVac and resuspended in 1% formic acid,

Following 1D PAGE separation, band excision, trypsin digestion and protein extraction, samples were analysed with an automated nanoLC MS/MS system. Samples were first separated using an easy nano-LC 1000 system (Thermo) with a 75um x 2cm reverse phased column (C18 3um 100 Å). Injection volumes of 2 µl were used with a flow rate of 300nl/min. The mobile phase consisted of solvents A (water with 0.1% formic acid) and B (ACN with 0.1% formic acid), and a linear gradient of 5% Solvent A to 40% Solvent B in 70 min then to 95% B in 5 min which remained for 15 min prior to column re-equilibration at 5% was used. This was coupled to an LTQ Orbitrap XL mass spectrometer equipped with a nanoelectrospray ionization source (ThermoFinnigan, San Jose, CA). The mass spectrometer was set up in a data-dependent mode for which every MS scan (m/z acquisition range from 300-1800) was followed by tandem (Collision induced dissociation (CID)) mass spectra scans (**m**/**z** acquisition range from 350-5000 Da) for the five most intense peaks in any full scan.

**Protein identification and database searching**

Tandem mass spectra were extracted by Proteome Discoverer version 1.3.0.339. Charge state deconvolution and deisotoping were not performed. All MS/MS samples were analyzed using Mascot (Matrix Science, London, UK; version 1.3.0.339). Mascot was set up to search Mascot5_swissprot_Homo sapiens (human) assuming the digestion enzyme trypsin. Mascot was searched with a fragment ion mass tolerance of 0.80 Da and a parent ion tolerance of 10.0 PPM. Trypsin/P was set as the protease, allowing for one missed cleavage cysteine, oxidation of methionine, carbamidomethylaltion of cysteine and phosphorylation of serine, threonine and tyrosine were specified in Mascot as variable modifications. The false discovery rate for automated interpretation of the MS/MS spectra was set between 0.01-0.05 when searched against a decoy database (a comparable database with all the protein sequences reversed). The abundance of an individual protein was calculated as the sum of the three most intense peptide precursor ions derived from that protein. The normalization of peak areas was achieved by correcting values for total protein concentrations determined for individual BAL samples using the bicinchoninic acid method [1] with further correction for the lavage dilution using the urea method [2]. The annotation of protein cellular localization and biological function were mapped to Gene Ontology (GO) biological process terms. Further to this, proteins were further excluded if not found present in at least 3 out of 5 subjects.

**Table s1: Alveolar proteins identified by 1D PAGE and nano LC MS/MS in healthy control lung lining fluids.** The 277 proteins identified in healthy control subjects investigated, (n=5), alongside the mean of the three most abundant ion peaks of each respective protein identified. Accession numbers included according to entries in UniProtKB/Swiss-Prot. Identified proteases, based on gene ontology are highlighted.

| **Accession Number** | **Proteins identified with MASCOT** | **Area (of three most intense precursor ions)** |
| --- | --- | --- |
| P02787 | Serotransferrin [Homo sapiens (Human)] - [TRFE_HUMAN] | 1.28E+10 |
| P01857 | Ig gamma-1 chain C region; [Homo sapiens (Human)] - [IGHG1_HUMAN] | 1.23E+10 |
| P01860 | Ig gamma-3 chain C region [Homo sapiens (Human)] - [IGHG3_HUMAN] | 9.36E+09 |
| P01859 | Ig gamma-2 chain C region; [Homo sapiens (Human)] - [IGHG2_HUMAN] | 9.10E+09 |
| P01861 | Ig gamma-4 chain C region; [Homo sapiens (Human)] - [IGHG4_HUMAN] | 9.10E+09 |
| P01834 | Ig kappa chain C region; [Homo sapiens (Human)] - [IGKC_HUMAN] | 5.90E+09 |
| Q5JPI3 | Uncharacterized protein C3orf38; [Homo sapiens (Human)] - [CC038_HUMAN] | 4.10E+09 |
| P02768 | Serum albumin; Flags: Precursor; [Homo sapiens (Human)] - [ALBU_HUMAN] | 3.97E+09 |
| P0CG05 | Ig lambda-2 chain C regions; [Homo sapiens (Human)] - [LAC2_HUMAN] | 3.07E+09 |
| P01876 | Ig alpha-1 chain C region; [Homo sapiens (Human)] - [IGHA1_HUMAN] | 2.81E+09 |
| P01009 | Alpha-1-antitrypsin; [Homo sapiens (Human)] - [A1AT_HUMAN] | 2.69E+09 |
| P01877 | Ig alpha-2 chain C region; [Homo sapiens (Human)] - [IGHA2_HUMAN] | 2.05E+09 |
| B9A064 | Immunoglobulin lambda-like polypeptide 5; [Homo sapiens (Human)] - [IGLL5_HUMAN] | 1.96E+09 |
| P01771 | Ig heavy chain V-III region HIL; [Homo sapiens (Human)] - [HV310_HUMAN] | 1.66E+09 |
| Q13616 | Cullin-1; Short=CUL-1; [Homo sapiens (Human)] - [CUL1_HUMAN] | 1.50E+09 |
| P02788 | Lactotransferrin; [Homo sapiens (Human)] - [TRFL_HUMAN] | 1.38E+09 |
| P52565 | Rho GDP-dissociation inhibitor 1 [Homo sapiens (Human)] - [GDIR1_HUMAN] | 1.36E+09 |
| P01764 | Ig heavy chain V-III region VH26; [Homo sapiens (Human)] - [HV303_HUMAN] | 1.25E+09 |
| P01768 | Ig heavy chain V-III region CAM; [Homo sapiens (Human)] - [HV307_HUMAN] | 1.22E+09 |
| P01766 | Ig heavy chain V-III region BRO; [Homo sapiens (Human)] - [HV305_HUMAN] | 1.19E+09 |
| P01779 | Ig heavy chain V-III region TUR; [Homo sapiens (Human)] - [HV318_HUMAN] | 1.18E+09 |
| P01781 | Ig heavy chain V-III region GAL; [Homo sapiens (Human)] - [HV320_HUMAN] | 1.17E+09 |
| P01767 | Ig heavy chain V-III region BUT; [Homo sapiens (Human)] - [HV306_HUMAN] | 1.16E+09 |
| P07339 | Cathepsin D; [Homo sapiens (Human)] - [CATD_HUMAN] | 1.10E+09 |
| Q8IVU3 | Probable E3 ubiquitin-protein ligase HERC6; [Homo sapiens (Human)] - [HERC6_HUMAN] | 8.68E+08 |
| P02790 | Hemopexin; [Homo sapiens (Human)] - [HEMO_HUMAN] | 7.70E+08 |
| P11684 | Uteroglobin; [Homo sapiens (Human)] - [UTER_HUMAN] | 6.27E+08 |
| P00450 | Ceruloplasmin; [Homo sapiens (Human)] - [CERU_HUMAN] | 5.37E+08 |
| P01833 | Polymeric immunoglobulin receptor; Short=PIgR; Short=Poly-Ig receptor; [Homo sapiens (Human)] - [PIGR_HUMAN] | 5.24E+08 |
| P78385 | Keratin, type II cuticular Hb3; [Homo sapiens (Human)] - [KRT83_HUMAN] | 5.19E+08 |
| P78386 | Keratin, type II cuticular Hb5; [Homo sapiens (Human)] - [KRT85_HUMAN] | 5.19E+08 |
| O43790 | Keratin, type II cuticular Hb6; [Homo sapiens (Human)] - [KRT86_HUMAN] | 5.19E+08 |
| P01611 | Ig kappa chain V-I region Wes; [Homo sapiens (Human)] - [KV119_HUMAN] | 5.03E+08 |
| P01597 | Ig kappa chain V-I region DEE; [Homo sapiens (Human)] - [KV105_HUMAN] | 4.84E+08 |
| P80362 | Ig kappa chain V-I region WAT; [Homo sapiens (Human)] - [KV125_HUMAN] | 4.74E+08 |
| P01715 | Ig lambda chain V-IV region Bau; [Homo sapiens (Human)] - [LV401_HUMAN] | 4.40E+08 |
| P01598 | Ig kappa chain V-I region EU; [Homo sapiens (Human)] - [KV106_HUMAN] | 4.22E+08 |
| A4UGR9 | Xin actin-binding repeat-containing protein 2; [Homo sapiens (Human)] - [XIRP2_HUMAN] | 4.22E+08 |
| P00738 | Haptoglobin; [Homo sapiens (Human)] - [HPT_HUMAN] | 3.82E+08 |
| P04433 | Ig kappa chain V-III region VG; [Homo sapiens (Human)] - [KV309_HUMAN] | 3.81E+08 |
| Q15323 | Keratin, type I cuticular Ha1; [Homo sapiens (Human)] - [K1H1_HUMAN] | 3.68E+08 |
| O76009 | Keratin, type I cuticular Ha3-I; [Homo sapiens (Human)] - [KT33A_HUMAN] | 3.68E+08 |
| Q14525 | Keratin, type I cuticular Ha3-II; [Homo sapiens (Human)] - [KT33B_HUMAN] | 3.68E+08 |
| O76011 | Keratin, type I cuticular Ha4; [Homo sapiens (Human)] - [KRT34_HUMAN] | 3.60E+08 |
| Q5J8X5 | Membrane-spanning 4-domains subfamily A member 13; [Homo sapiens (Human)] - [M4A13_HUMAN] | 3.33E+08 |
| P01620 | Ig kappa chain V-III region SIE; [Homo sapiens (Human)] - [KV302_HUMAN] | 3.17E+08 |
| P01624 | Ig kappa chain V-III region POM; [Homo sapiens (Human)] - [KV306_HUMAN] | 3.13E+08 |
| P06309 | Ig kappa chain V-II region GM607; [Homo sapiens (Human)] - [KV205_HUMAN] | 2.91E+08 |
| P01717 | Ig lambda chain V-IV region Hil; [Homo sapiens (Human)] - [LV403_HUMAN] | 2.91E+08 |
| Q9NSB4 | Keratin, type II cuticular Hb2; [Homo sapiens (Human)] - [KRT82_HUMAN] | 2.86E+08 |
| P80419 | Ig heavy chain V-III region GAR; [Homo sapiens (Human)] - [HV322_HUMAN] | 2.84E+08 |
| P80748 | Ig lambda chain V-III region LOI; [Homo sapiens (Human)] - [LV302_HUMAN] | 2.61E+08 |
| Q96PZ0 | Pseudouridylate synthase 7 homolog; [Homo sapiens (Human)] - [PUS7_HUMAN] | 2.57E+08 |
| P01609 | Ig kappa chain V-I region Scw; [Homo sapiens (Human)] - [KV117_HUMAN] | 2.48E+08 |
| Q9BX69 | Caspase recruitment domain-containing protein 6; [Homo sapiens (Human)] - [CARD6_HUMAN] | 2.40E+08 |
| P01622 | Ig kappa chain V-III region Ti; [Homo sapiens (Human)] - [KV304_HUMAN] | 2.35E+08 |
| Q14532 | Keratin, type I cuticular Ha2; [Homo sapiens (Human)] - [K1H2_HUMAN] | 2.25E+08 |
| A6NJ16 | Putative V-set and immunoglobulin domain-containing protein 6; Flags: [Homo sapiens (Human)] - [VSIG6_HUMAN] | 2.22E+08 |
| P04264 | Keratin, type II cytoskeletal 1; [Homo sapiens (Human)] - [K2C1_HUMAN] | 2.21E+08 |
| Q9UNN5 | FAS-associated factor 1; Short=hFAF1; [Homo sapiens (Human)] - [FAF1_HUMAN] | 2.21E+08 |
| P02774 | Vitamin D-binding protein; [Homo sapiens (Human)] - [VTDB_HUMAN] | 2.19E+08 |
| P01700 | Ig lambda chain V-I region HA; [Homo sapiens (Human)] - [LV102_HUMAN] | 2.17E+08 |
| P01743 | Ig heavy chain V-I region HG3; [Homo sapiens (Human)] - [HV102_HUMAN] | 2.14E+08 |
| P68871 | Hemoglobin subunit beta; [Homo sapiens (Human)] - [HBB_HUMAN] | 2.09E+08 |
| P69905 | Hemoglobin subunit alpha; [Homo sapiens (Human)] - [HBA_HUMAN] | 2.06E+08 |
| P02763 | Alpha-1-acid glycoprotein 1; [Homo sapiens (Human)] - [A1AG1_HUMAN] | 2.04E+08 |
| Q96QB1 | Rho GTPase-activating protein 7; [Homo sapiens (Human)] - [RHG07_HUMAN] | 2.02E+08 |
| P01608 | RecName: Full=Ig kappa chain V-I region Roy; [Homo sapiens (Human)] - [KV116_HUMAN] | 1.96E+08 |
| Q8IWL1 | Pulmonary surfactant-associated protein A2; [Homo sapiens (Human)] - [SFPA2_HUMAN] | 1.95E+08 |
| P13647 | Keratin, type II cytoskeletal 5; [Homo sapiens (Human)] - [K2C5_HUMAN] | 1.88E+08 |
| P02538 | Keratin, type II cytoskeletal 6A; [Homo sapiens (Human)] - [K2C6A_HUMAN] | 1.88E+08 |
| P04259 | Keratin, type II cytoskeletal 6B; [Homo sapiens (Human)] - [K2C6B_HUMAN] | 1.88E+08 |
| P19652 | Alpha-1-acid glycoprotein 2; Short=AGP 2; [Homo sapiens (Human)] - [A1AG2_HUMAN] | 1.81E+08 |
| P01621 | Ig kappa chain V-III region NG9; [Homo sapiens (Human)] - [KV303_HUMAN] | 1.74E+08 |
| P02042 | Hemoglobin subunit delta; [Homo sapiens (Human)] - [HBD_HUMAN] | 1.66E+08 |
| P01625 | Ig kappa chain V-IV region Len; [Homo sapiens (Human)] - [KV402_HUMAN] | 1.66E+08 |
| Q92764 | Keratin, type I cuticular Ha5; [Homo sapiens (Human)] - [KRT35_HUMAN] | 1.62E+08 |
| P15924 | Desmoplakin; Short=DP; [Homo sapiens (Human)] - [DESP_HUMAN] | 1.60E+08 |
| P35908 | Keratin, type II cytoskeletal 2 epidermal; [Homo sapiens (Human)] - [K22E_HUMAN] | 1.50E+08 |
| P01011 | Alpha-1-antichymotrypsin; Short=ACT; [Homo sapiens (Human)] - [AACT_HUMAN] | 1.49E+08 |
| O75818 | Ribonuclease P protein subunit p40; Short=RNaseP protein p40; EC=3.1.26.5; [Homo sapiens (Human)] - [RPP40_HUMAN] | 1.42E+08 |
| Q8TCU6 | Phosphatidylinositol 3,4,5-trisphosphate-dependent Rac exchanger 1 protein; Short=P-Rex1; Short=PtdIns(3,4,5)-dependent Rac exchanger 1; [Homo sapiens (Human)] - [PREX1_HUMAN] | 1.41E+08 |
| P04217 | Alpha-1B-glycoprotein; [Homo sapiens (Human)] - [A1BG_HUMAN] | 1.36E+08 |
| P35527 | Keratin, type I cytoskeletal 9; [Homo sapiens (Human)] - [K1C9_HUMAN] | 1.30E+08 |
| P04208 | Ig lambda chain V-I region WAH; [Homo sapiens (Human)] - [LV106_HUMAN] | 1.26E+08 |
| P13645 | Keratin, type I cytoskeletal 10; [Homo sapiens (Human)] - [K1C10_HUMAN] | 1.23E+08 |
| O76074 | cGMP-specific 3',5'-cyclic phosphodiesterase; EC=3.1.4.35; [Homo sapiens (Human)] - [PDE5A_HUMAN] | 1.22E+08 |
| P61769 | Beta-2-microglobulin; Contains: RecName: Full=Beta-2-microglobulin form pI 5.3; [Homo sapiens (Human)] - [B2MG_HUMAN] | 1.20E+08 |
| P25311 | Zinc-alpha-2-glycoprotein; Short=Zn-alpha-2-GP; Short=Zn-alpha-2-glycoprotein; [Homo sapiens (Human)] - [ZA2G_HUMAN] | 1.16E+08 |
| P01613 | Ig kappa chain V-I region Ni; [Homo sapiens (Human)] - [KV121_HUMAN] | 1.15E+08 |
| P01825 | Ig heavy chain V-II region NEWM; [Homo sapiens (Human)] - [HV207_HUMAN] | 1.14E+08 |
| Q8WWI1 | LIM domain only protein 7; Short=LMO-7; [Homo sapiens (Human)] - [LMO7_HUMAN] | 1.14E+08 |
| P02766 | Transthyretin; [Homo sapiens (Human)] - [TTHY_HUMAN] | 1.12E+08 |
| P61960 | Ubiquitin-fold modifier 1; [Homo sapiens (Human)] - [UFM1_HUMAN] | 1.08E+08 |
| O60763 | General vesicular transport factor p115; [Homo sapiens (Human)] - [USO1_HUMAN] | 1.08E+08 |
| P31946 | 14-3-3 protein beta/alpha; [Homo sapiens (Human)] - [1433B_HUMAN] | 1.07E+08 |
| Q04917 | 14-3-3 protein eta; AltName: Full=Protein AS1; [Homo sapiens (Human)] - [1433F_HUMAN] | 1.07E+08 |
| P61981 | 14-3-3 protein gamma; [Homo sapiens (Human)] - [1433G_HUMAN] | 1.07E+08 |
| P63104 | 14-3-3 protein zeta/delta; [Homo sapiens (Human)] - [1433Z_HUMAN] | 1.07E+08 |
| P04211 | Ig lambda chain V region 4A; [Homo sapiens (Human)] - [LV001_HUMAN] | 1.06E+08 |
| P20929 | Nebulin; [Homo sapiens (Human)] - [NEBU_HUMAN] | 1.06E+08 |
| P02533 | Keratin, type I cytoskeletal 14; [Homo sapiens (Human)] - [K1C14_HUMAN] | 1.05E+08 |
| P08779 | Keratin, type I cytoskeletal 16; [Homo sapiens (Human)] - [K1C16_HUMAN] | 1.05E+08 |
| P01019 | Angiotensinogen; [Homo sapiens (Human)] - [ANGT_HUMAN] | 9.61E+07 |
| P02647 | Apolipoprotein A-I; Short=Apo-AI; Short=ApoA-I; [Homo sapiens (Human)] - [APOA1_HUMAN] | 9.59E+07 |
| P06703 | Protein S100-A6; [Homo sapiens (Human)] - [S10A6_HUMAN] | 9.49E+07 |
| P06313 | Ig kappa chain V-IV region JI; Flags: [Homo sapiens (Human)] - [KV403_HUMAN] | 9.06E+07 |
| P62258 | 14-3-3 protein epsilon; Short=14-3-3E; [Homo sapiens (Human)] - [1433E_HUMAN] | 9.02E+07 |
| Q8TF72 | Protein Shroom3; [Homo sapiens (Human)] - [SHRM3_HUMAN] | 8.34E+07 |
| P01591 | Immunoglobulin J chain; Flags: [Homo sapiens (Human)] - [IGJ_HUMAN] | 8.29E+07 |
| Q8NEZ4 | Histone-lysine N-methyltransferase MLL3; EC=2.1.1.43; [Homo sapiens (Human)] - [MLL3_HUMAN] | 7.98E+07 |
| Q3L8U1 | Chromodomain-helicase-DNA-binding protein 9; Short=CHD-9; EC=3.6.4.12 [Homo sapiens (Human)] - [CHD9_HUMAN] | 7.91E+07 |
| O94913 | Pre-mRNA cleavage complex 2 protein Pcf11; [Homo sapiens (Human)] - [PCF11_HUMAN] | 7.88E+07 |
| P16070 | CD44 antigen; [Homo sapiens (Human)] - [CD44_HUMAN] | 7.78E+07 |
| P06331 | Ig heavy chain V-II region ARH-77; Flags: [Homo sapiens (Human)] - [HV209_HUMAN] | 7.57E+07 |
| P02765 | Alpha-2-HS-glycoprotein; [Homo sapiens (Human)] - [FETUA_HUMAN] | 7.52E+07 |
| Q9BYR6 | Keratin-associated protein 3-3; [Homo sapiens (Human)] - [KRA33_HUMAN] | 7.01E+07 |
| P01702 | Ig lambda chain V-I region NIG-64; [Homo sapiens (Human)] - [LV104_HUMAN] | 6.96E+07 |
| Q09428 | ATP-binding cassette sub-family C member 8; [Homo sapiens (Human)] - [ABCC8_HUMAN] | 6.85E+07 |
| Q494V2 | Coiled-coil domain-containing protein 37; [Homo sapiens (Human)] - [CCD37_HUMAN] | 6.84E+07 |
| P16401 | Histone H1.5; [Homo sapiens (Human)] - [H15_HUMAN] | 6.82E+07 |
| P01814 | Ig heavy chain V-II region OU; [Homo sapiens (Human)] - [HV201_HUMAN] | 6.80E+07 |
| P01023 | Alpha-2-macroglobulin; [Homo sapiens (Human)] - [A2MG_HUMAN] | 6.75E+07 |
| P08758 | Annexin A5; [Homo sapiens (Human)] - [ANXA5_HUMAN] | 6.60E+07 |
| P01871 | Ig mu chain C region; [Homo sapiens (Human)] - [IGHM_HUMAN] | 6.47E+07 |
| P23083 | Ig heavy chain V-I region V35; Flags: Precursor; [Homo sapiens (Human)] - [HV103_HUMAN] | 6.05E+07 |
| O94808 | Glucosamine--fructose-6-phosphate aminotransferase [isomerizing] 2; EC=2.6.1.16; [Homo sapiens (Human)] - [GFPT2_HUMAN] | 5.95E+07 |
| P09210 | Glutathione S-transferase A2; EC=2.5.1.18; [Homo sapiens (Human)] - [GSTA2_HUMAN] | 5.83E+07 |
| P08118 | Beta-microseminoprotein; Precursor; [Homo sapiens (Human)] - [MSMB_HUMAN] | 5.82E+07 |
| Q9UGM3 | Deleted in malignant brain tumors 1 protein; [Homo sapiens (Human)] - [DMBT1_HUMAN] | 5.59E+07 |
| P60709 | Actin, cytoplasmic 1; [Homo sapiens (Human)] - [ACTB_HUMAN] | 5.55E+07 |
| P61916 | Epididymal secretory protein E1; [Homo sapiens (Human)] - [NPC2_HUMAN] | 5.26E+07 |
| P08185 | Corticosteroid-binding globulin; Short=CBG; [Homo sapiens (Human)] - [CBG_HUMAN] | 5.17E+07 |
| Q9BYN8 | 28S ribosomal protein S26, mitochondrial; Short=MRP-S26; Short=S26mt; [Homo sapiens (Human)] - [RT26_HUMAN] | 4.89E+07 |
| Q66K66 | Transmembrane protein 198; [Homo sapiens (Human)] - [TM198_HUMAN] | 4.77E+07 |
| P62805 | Histone H4; [Homo sapiens (Human)] - [H4_HUMAN] | 4.75E+07 |
| P35247 | Pulmonary surfactant-associated protein D; Short=PSP-D; Short=SP-D; [Homo sapiens (Human)] - [SFTPD_HUMAN] | 4.71E+07 |
| Q9HD64 | G antigen family D member 2; [Homo sapiens (Human)] - [GAGD2_HUMAN] | 4.41E+07 |
| P05362 | Intercellular adhesion molecule 1; Short=ICAM-1; [Homo sapiens (Human)] - [ICAM1_HUMAN] | 4.01E+07 |
| Q99497 | Protein DJ-1; EC=3.4 [Homo sapiens (Human)] - [PARK7_HUMAN] | 3.98E+07 |
| P60174 | Triosephosphate isomerase; Short=TIM; EC=5.3.1.1; [Homo sapiens (Human)] - [TPIS_HUMAN] | 3.83E+07 |
| P25098 | Beta-adrenergic receptor kinase 1; Short=Beta-ARK-1; EC=2.7.11.15; [Homo sapiens (Human)] - [ARBK1_HUMAN] | 3.66E+07 |
| P62158 | Calmodulin; Short=CaM; [Homo sapiens (Human)] - [CALM_HUMAN] | 3.58E+07 |
| Q14019 | Coactosin-like protein; [Homo sapiens (Human)] - [COTL1_HUMAN] | 3.46E+07 |
| Q5TH69 | Brefeldin A-inhibited guanine nucleotide-exchange protein 3; [Homo sapiens (Human)] - [BIG3_HUMAN] | 3.44E+07 |
| P09211 | Glutathione S-transferase P; EC=2.5.1.18 [Homo sapiens (Human)] - [GSTP1_HUMAN] | 3.42E+07 |
| Q14CN4 | Keratin, type II cytoskeletal 72; [Homo sapiens (Human)] - [K2C72_HUMAN] | 3.38E+07 |
| Q5VU13 | V-set and immunoglobulin domain-containing protein 8; [Homo sapiens (Human)] - [VSIG8_HUMAN] | 3.05E+07 |
| P29508 | Serpin B3; AltName: Full=Protein T4-A; [Homo sapiens (Human)] - [SPB3_HUMAN] | 3.01E+07 |
| P01699 | Ig lambda chain V-I region VOR; [Homo sapiens (Human)] - [LV101_HUMAN] | 2.97E+07 |
| P01008 | Antithrombin-III; Short=ATIII; [Homo sapiens (Human)] - [ANT3_HUMAN] | 2.92E+07 |
| P12821 | Angiotensin-converting enzyme; Short=ACE; EC=3.2.1.-; EC=3.4.15.1; AltName: Full=Dipeptidyl carboxypeptidase I; [Homo sapiens (Human)] - [ACE_HUMAN] | 2.86E+07 |
| P01714 | Ig lambda chain V-III region SH; [Homo sapiens (Human)] - [LV301_HUMAN] | 2.83E+07 |
| Q96CB9 | Putative methyltransferase NSUN4; EC=2.1.1.-; [Homo sapiens (Human)] - [NSUN4_HUMAN] | 2.80E+07 |
| P09668 | Pro-cathepsin H; [Homo sapiens (Human)] - [CATH_HUMAN] | 2.79E+07 |
| P02750 | Leucine-rich alpha-2-glycoprotein; Short=LRG; [Homo sapiens (Human)] - [A2GL_HUMAN] | 2.66E+07 |
| P02652 | Apolipoprotein A-II; [Homo sapiens (Human)] - [APOA2_HUMAN] | 2.59E+07 |
| P06317 | Ig lambda chain V-VI region SUT; [Homo sapiens (Human)] - [LV603_HUMAN] | 2.58E+07 |
| P43652 | Afamin [Homo sapiens (Human)] - [AFAM_HUMAN] | 2.55E+07 |
| P07988 | Pulmonary surfactant-associated protein B; [Homo sapiens (Human)] - [PSPB_HUMAN] | 2.54E+07 |
| P09525 | Annexin A4; [Homo sapiens (Human)] - [ANXA4_HUMAN] | 2.52E+07 |
| Q9BYR8 | Keratin-associated protein 3-1; [Homo sapiens (Human)] - [KRA31_HUMAN] | 2.51E+07 |
| Q13228 | Selenium-binding protein 1; [Homo sapiens (Human)] - [SBP1_HUMAN] | 2.43E+07 |
| P00915 | Carbonic anhydrase 1; EC=4.2.1.1; [Homo sapiens (Human)] - [CAH1_HUMAN] | 2.36E+07 |
| P37837 | Transaldolase; EC=2.2.1.2; [Homo sapiens (Human)] - [TALDO_HUMAN] | 2.33E+07 |
| Q86VB7 | Scavenger receptor cysteine-rich type 1 protein M130; [Homo sapiens (Human)] - [C163A_HUMAN] | 2.29E+07 |
| P21926 | CD9 antigen; [Homo sapiens (Human)] - [CD9_HUMAN] | 2.20E+07 |
| O94830 | Phospholipase DDHD2; [Homo sapiens (Human)] - [DDHD2_HUMAN] | 2.13E+07 |
| Q9Y4R8 | Telomere length regulation protein TEL2 homolog; [Homo sapiens (Human)] - [TELO2_HUMAN] | 1.81E+07 |
| P0C0L4 | Complement C4-A; [Homo sapiens (Human)] - [CO4A_HUMAN] | 1.80E+07 |
| Q9NR61 | Delta-like protein 4; [Homo sapiens (Human)] - [DLL4_HUMAN] | 1.80E+07 |
| Q13421 | Mesothelin; [Homo sapiens (Human)] - [MSLN_HUMAN] | 1.78E+07 |
| Q6IQ23 | Pleckstrin homology domain-containing family A member 7; Short=PH domain-containing family A member 7; [Homo sapiens (Human)] - [PKHA7_HUMAN] | 1.78E+07 |
| P27487 | Dipeptidyl peptidase 4; [Homo sapiens (Human)] - [DPP4_HUMAN] | 1.64E+07 |
| P61160 | Actin-related protein 2; [Homo sapiens (Human)] - [ARP2_HUMAN] | 1.56E+07 |
| Q9Y2F5 | Uncharacterized protein KIAA0947; [Homo sapiens (Human)] - [K0947_HUMAN] | 1.56E+07 |
| P05543 | Thyroxine-binding globulin; [Homo sapiens (Human)] - [THBG_HUMAN] | 1.56E+07 |
| Q6P5S2 | UPF0762 protein C6orf58; [Homo sapiens (Human)] - [CF058_HUMAN] | 1.50E+07 |
| P08174 | Complement decay-accelerating factor; [Homo sapiens (Human)] - [DAF_HUMAN] | 1.49E+07 |
| P42330 | Aldo-keto reductase family 1 member C3; EC=1.-.-.-; [Homo sapiens (Human)] - [AK1C3_HUMAN] | 1.46E+07 |
| Q9H5K3 | Protein kinase-like protein SgK196; [Homo sapiens (Human)] - [SG196_HUMAN] | 1.39E+07 |
| P15941 | Mucin-1; Short=MUC-1; [Homo sapiens (Human)] - [MUC1_HUMAN] | 1.31E+07 |
| P10599 | Thioredoxin; Short=Trx; [Homo sapiens (Human)] - [THIO_HUMAN] | 1.30E+07 |
| Q8NFJ5 | Retinoic acid-induced protein 3; [Homo sapiens (Human)] - [RAI3_HUMAN] | 1.29E+07 |
| P01024 | Complement C3; [Homo sapiens (Human)] - [CO3_HUMAN] | 1.25E+07 |
| P48065 | Sodium- and chloride-dependent betaine transporter; [Homo sapiens (Human)] - [S6A12_HUMAN] | 1.25E+07 |
| Q99879 | Histone H2B type 1-M; [Homo sapiens (Human)] - [H2B1M_HUMAN] | 1.23E+07 |
| P33764 | Protein S100-A3; [Homo sapiens (Human)] - [S10A3_HUMAN] | 1.22E+07 |
| P62937 | Peptidyl-prolyl cis-trans isomerase A; Short=PPIase A; EC=5.2.1.8; [Homo sapiens (Human)] - [PPIA_HUMAN] | 1.19E+07 |
| P43251 | Biotinidase; Short=Biotinase; EC=3.5.1.12; [Homo sapiens (Human)] - [BTD_HUMAN] | 1.19E+07 |
| P12955 | Xaa-Pro dipeptidase; Short=X-Pro dipeptidase; EC=3.4.13.9; [Homo sapiens (Human)] - [PEPD_HUMAN] | 1.18E+07 |
| Q08380 | Galectin-3-binding protein; [Homo sapiens (Human)] - [LG3BP_HUMAN] | 1.14E+07 |
| Q1ED39 | Protein C16orf88; AltName: Full=Testis-specific gene 118 protein; [Homo sapiens (Human)] - [CP088_HUMAN] | 1.13E+07 |
| P55786 | Puromycin-sensitive aminopeptidase; Short=PSA; EC=3.4.11.14; AltName: Full=Cytosol alanyl aminopeptidase; Short=AAP-S; [Homo sapiens (Human)] - [PSA_HUMAN] | 1.13E+07 |
| Q9H299 | SH3 domain-binding glutamic acid-rich-like protein 3; AltName: Full=SH3 domain-binding protein 1; Short=SH3BP-1; [Homo sapiens (Human)] - [SH3L3_HUMAN] | 1.13E+07 |
| O75882 | Attractin; AltName: Full=DPPT-L; AltName: Full=Mahogany homolog; Flags: Precursor; [Homo sapiens (Human)] - [ATRN_HUMAN] | 1.13E+07 |
| P52566 | Rho GDP-dissociation inhibitor 2; Short=Rho GDI 2; AltName: Full=Ly-GDI; AltName: Full=Rho-GDI beta; [Homo sapiens (Human)] - [GDIR2_HUMAN] | 1.12E+07 |
| Q6NXT2 | Histone H3.3C; [Homo sapiens (Human)] - [H3C_HUMAN] | 1.08E+07 |
| Q8N490 | Probable hydrolase PNKD; EC=3.-.-.-; [Homo sapiens (Human)] - [PNKD_HUMAN] | 1.07E+07 |
| P06727 | Apolipoprotein A-IV; Short=Apo-AIV; Short=ApoA-IV; [Homo sapiens (Human)] - [APOA4_HUMAN] | 1.04E+07 |
| P06396 | Gelsolin; [Homo sapiens (Human)] - [GELS_HUMAN] | 9.95E+06 |
| P05155 | Plasma protease C1 inhibitor; Short=C1 Inh; Short=C1Inh; [Homo sapiens (Human)] - [IC1_HUMAN] | 9.83E+06 |
| P15090 | Fatty acid-binding protein, adipocyte; [Homo sapiens (Human)] - [FABP4_HUMAN] | 9.73E+06 |
| Q9BZL6 | Serine/threonine-protein kinase D2; EC=2.7.11.13; AltName: Full=nPKC-D2; [Homo sapiens (Human)] - [KPCD2_HUMAN] | 9.67E+06 |
| P01719 | Ig lambda chain V-V region DEL; [Homo sapiens (Human)] - [LV501_HUMAN] | 9.50E+06 |
| P00918 | Carbonic anhydrase 2; EC=4.2.1.1; [Homo sapiens (Human)] - [CAH2_HUMAN] | 9.50E+06 |
| P00441 | Superoxide dismutase [Cu-Zn]; EC=1.15.1.1; [Homo sapiens (Human)] - [SODC_HUMAN] | 9.13E+06 |
| P40199 | Carcinoembryonic antigen-related cell adhesion molecule 6; [Homo sapiens (Human)] - [CEAM6_HUMAN] | 9.07E+06 |
| Q8WWI5 | Choline transporter-like protein 1; [Homo sapiens (Human)] - [CTL1_HUMAN] | 8.96E+06 |
| O14607 | Histone demethylase UTY; EC=1.14.11.-; [Homo sapiens (Human)] - [UTY_HUMAN] | 8.83E+06 |
| Q86TJ2 | Transcriptional adapter 2-beta; [Homo sapiens (Human)] - [TAD2B_HUMAN] | 8.54E+06 |
| Q9BYR4 | Keratin-associated protein 4-3; [Homo sapiens (Human)] - [KRA43_HUMAN] | 8.45E+06 |
| P30740 | Leukocyte elastase inhibitor; Short=LEI; [Homo sapiens (Human)] - [ILEU_HUMAN] | 8.21E+06 |
| Q9Y279 | V-set and immunoglobulin domain-containing protein 4; [Homo sapiens (Human)] - [VSIG4_HUMAN] | 8.17E+06 |
| P0CG48 | Polyubiquitin-C; Contains: [Homo sapiens (Human)] - [UBC_HUMAN] | 7.82E+06 |
| P20142 | Gastricsin; EC=3.4.23.3; [Homo sapiens (Human)] - [PEPC_HUMAN] | 7.66E+06 |
| P31949 | Protein S100-A11; [Homo sapiens (Human)] - [S10AB_HUMAN] | 7.59E+06 |
| P04080 | Cystatin-B; [Homo sapiens (Human)] - [CYTB_HUMAN] | 7.58E+06 |
| Q9BY67 | Cell adhesion molecule 1; [Homo sapiens (Human)] - [CADM1_HUMAN] | 7.37E+06 |
| P04229 | HLA class II histocompatibility antigen, DRB1-1 beta chain; [Homo sapiens (Human)] - [2B11_HUMAN] | 7.37E+06 |
| Q8TDL5 | Long palate, lung and nasal epithelium carcinoma-associated protein 1; [Homo sapiens (Human)] - [LPLC1_HUMAN] | 7.16E+06 |
| Q96KK5 | Histone H2A type 1-H; [Homo sapiens (Human)] - [H2A1H_HUMAN] | 7.01E+06 |
| P78417 | Glutathione S-transferase omega-1; Short=GSTO-1; EC=2.5.1.18; [Homo sapiens (Human)] - [GSTO1_HUMAN] | 6.98E+06 |
| P06681 | Complement C2; EC=3.4.21.43; [Homo sapiens (Human)] - [CO2_HUMAN] | 6.82E+06 |
| Q6UXN9 | WD repeat-containing protein 82; [Homo sapiens (Human)] - [WDR82_HUMAN] | 6.67E+06 |
| P01880 | Ig delta chain C region; [Homo sapiens (Human)] - [IGHD_HUMAN] | 6.39E+06 |
| P81605 | Dermcidin; [Homo sapiens (Human)] - [DCD_HUMAN] | 6.20E+06 |
| P30044 | Peroxiredoxin-5, mitochondrial; EC=1.11.1.15; [Homo sapiens (Human)] - [PRDX5_HUMAN] | 6.12E+06 |
| P07225 | Vitamin K-dependent protein S; [Homo sapiens (Human)] - [PROS_HUMAN] | 6.05E+06 |
| Q9Y624 | Junctional adhesion molecule A; Short=JAM-A; [Homo sapiens (Human)] - [JAM1_HUMAN] | 5.60E+06 |
| O14990 | Putative type-1 protein phosphatase inhibitor 4; Short=I-4; [Homo sapiens (Human)] - [IPP4_HUMAN] | 5.36E+06 |
| Q9Y5Z4 | Heme-binding protein 2; [Homo sapiens (Human)] - [HEBP2_HUMAN] | 5.28E+06 |
| Q9HC38 | Glyoxalase domain-containing protein 4; [Homo sapiens (Human)] - [GLOD4_HUMAN] | 5.28E+06 |
| P01042 | Kininogen-1; [Homo sapiens (Human)] - [KNG1_HUMAN] | 5.17E+06 |
| Q6A163 | Keratin, type I cytoskeletal 39; [Homo sapiens (Human)] - [K1C39_HUMAN] | 4.92E+06 |
| P41222 | Prostaglandin-H2 D-isomerase; EC=5.3.99.2; [Homo sapiens (Human)] - [PTGDS_HUMAN] | 4.69E+06 |
| Q86YZ3 | Hornerin; [Homo sapiens (Human)] - [HORN_HUMAN] | 4.56E+06 |
| P02144 | Myoglobin; [Homo sapiens (Human)] - [MYG_HUMAN] | 4.44E+06 |
| O00560 | Syntenin-1; [Homo sapiens (Human)] - [SDCB1_HUMAN] | 4.39E+06 |
| Q9UKY3 | Putative inactive carboxylesterase 4; [Homo sapiens (Human)] - [CES1P_HUMAN] | 4.34E+06 |
| P80188 | Neutrophil gelatinase-associated lipocalin; [Homo sapiens (Human)] - [NGAL_HUMAN] | 4.22E+06 |
| P22352 | Glutathione peroxidase 3; Short=GPx-3; Short=GSHPx-3; EC=1.11.1.9; [Homo sapiens (Human)] - [GPX3_HUMAN] | 4.07E+06 |
| P13987 | CD59 glycoprotein; [Homo sapiens (Human)] - [CD59_HUMAN] | 4.04E+06 |
| P61956 | Small ubiquitin-related modifier 2; Short=SUMO-2; [Homo sapiens (Human)] - [SUMO2_HUMAN] | 4.03E+06 |
| P30086 | Phosphatidylethanolamine-binding protein 1; Short=PEBP-1; [Homo sapiens (Human)] - [PEBP1_HUMAN] | 4.01E+06 |
| Q9UBR2 | Cathepsin Z; EC=3.4.18.1; [Homo sapiens (Human)] - [CATZ_HUMAN] | 4.01E+06 |
| P18669 | Phosphoglycerate mutase 1; EC=3.1.3.13; EC=5.4.2.1; EC=5.4.2.4; [Homo sapiens (Human)] - [PGAM1_HUMAN] | 3.87E+06 |
| Q9NSI2 | Uncharacterized protein C21orf70; [Homo sapiens (Human)] - [CU070_HUMAN] | 3.85E+06 |
| P01034 | Cystatin-C; [Homo sapiens (Human)] - [CYTC_HUMAN] | 3.83E+06 |
| Q9C099 | Leucine-rich repeat and coiled-coil domain-containing protein 1; [Homo sapiens (Human)] - [LRCC1_HUMAN] | 3.79E+06 |
| Q8N998 | Coiled-coil domain-containing protein 89; [Homo sapiens (Human)] - [CCD89_HUMAN] | 3.68E+06 |
| Q9BRA2 | Thioredoxin domain-containing protein 17; [Homo sapiens (Human)] - [TXD17_HUMAN] | 3.64E+06 |
| Q14671 | Pumilio homolog 1; Short=HsPUM; Short=Pumilio-1; [Homo sapiens (Human)] - [PUM1_HUMAN] | 3.58E+06 |
| P35237 | Serpin B6; [Homo sapiens (Human)] - [SPB6_HUMAN] | 3.30E+06 |
| Q05315 | Eosinophil lysophospholipase; EC=3.1.1.5; [Homo sapiens (Human)] - [LPPL_HUMAN] | 3.22E+06 |
| P15328 | Folate receptor alpha; Short=FR-alpha; [Homo sapiens (Human)] - [FOLR1_HUMAN] | 3.11E+06 |
| A6NKL6 | Transmembrane protein 200C; [Homo sapiens (Human)] - [T200C_HUMAN] | 2.87E+06 |
| O75368 | SH3 domain-binding glutamic acid-rich-like protein; [Homo sapiens (Human)] - [SH3L1_HUMAN] | 2.80E+06 |
| Q96C23 | Aldose 1-epimerase; EC=5.1.3.3; AltName: Full=Galactose mutarotase; [Homo sapiens (Human)] - [GALM_HUMAN] | 2.77E+06 |
| P61626 | Lysozyme C; EC=3.2.1.17; [Homo sapiens (Human)] - [LYSC_HUMAN] | 2.75E+06 |
| P00367 | Glutamate dehydrogenase 1, mitochondrial; Short=GDH 1; EC=1.4.1.3; Flags: Precursor; [Homo sapiens (Human)] - [DHE3_HUMAN] | 2.72E+06 |
| O75900 | Matrix metalloproteinase-23; Short=MMP-23; EC=3.4.24.-; [Homo sapiens (Human)] - [MMP23_HUMAN] | 2.66E+06 |
| Q16651 | Prostasin; EC=3.4.21.-; [Homo sapiens (Human)] - [PRSS8_HUMAN] | 2.41E+06 |
| Q5T750 | Skin-specific protein 32; [Homo sapiens (Human)] - [XP32_HUMAN] | 2.40E+06 |
| Q8TEK3 | Histone-lysine N-methyltransferase, H3 lysine-79 specific; EC=2.1.1.43; [Homo sapiens (Human)] - [DOT1L_HUMAN] | 2.39E+06 |
| O96009 | Napsin-A; [Homo sapiens (Human)] - [NAPSA_HUMAN] | 2.37E+06 |
| P30041 | Peroxiredoxin-6; EC=1.11.1.15; [Homo sapiens (Human)] - [PRDX6_HUMAN] | 2.13E+06 |
| P25815 | Protein S100-P; [Homo sapiens (Human)] - [S100P_HUMAN] | 2.12E+06 |
| Q9BZG2 | Testicular acid phosphatase; EC=3.1.3.2; [Homo sapiens (Human)] - [PPAT_HUMAN] | 2.01E+06 |
| Q7Z5L0 | Vitelline membrane outer layer protein 1 homolog; [Homo sapiens (Human)] - [VMO1_HUMAN] | 1.89E+06 |
| P17174 | Aspartate aminotransferase, cytoplasmic; EC=2.6.1.1; [Homo sapiens (Human)] - [AATC_HUMAN] | 1.80E+06 |
| P59665 | Neutrophil defensin 1; [Homo sapiens (Human)] - [DEF1_HUMAN] | 1.79E+06 |
| P02747 | Complement C1q subcomponent subunit C; [Homo sapiens (Human)] - [C1QC_HUMAN] | 1.71E+06 |
| Q5VWP2 | Protein FAM46C; [Homo sapiens (Human)] - [FA46C_HUMAN] | 1.33E+06 |
| O75223 | Gamma-glutamylcyclotransferase; [Homo sapiens (Human)] - [GGCT_HUMAN] | 1.29E+06 |
| O95436 | Sodium-dependent phosphate transport protein 2B; Short=Sodium-phosphate transport protein 2B [Homo sapiens (Human)] - [NPT2B_HUMAN] | 1.14E+06 |
| P68104 | Elongation factor 1-alpha 1; Short=EF-1-alpha-1; AltName: Full=Elongation factor Tu; Short=EF-Tu; [Homo sapiens (Human)] - [EF1A1_HUMAN] | 5.94E+05 |
| P53634 | Dipeptidyl peptidase 1; [Homo sapiens (Human)] – [CATC_HUMAN] | Present-not quantified |
| P09960 | Leukotriene A-4 hydrolase; [Homo sapiens (Human)] – [CATC_HUMAN] | Present-not quantified |

## 6.2 Supplementary references

1. Smith, P.K., et al., *Measurement of Protein Using Bicinchoninic Acid.* Analytical Biochemistry, 1985. **150**(1): p. 76-85.

2. van der Vliet, A., et al., *Determination of low-molecular-mass antioxidant concentrations in human respiratory tract lining fluids.* American Journal of Physiology-Lung Cellular and Molecular Physiology, 1999. **276**(2): p. L289-L296.
